# Effects of the powerball® system on muscle strength, coordination, fatigue, functionality and quality of life in people with multiple sclerosis. A randomized clinical trial

**DOI:** 10.1186/s12984-024-01325-w

**Published:** 2024-03-02

**Authors:** Aitor Blázquez-Fernández, Gemma López-Hazas-Jiménez, Diego Fernández-Vázquez, Víctor Navarro-López, Pilar Fernández-González, Selena Marcos-Antón, Francisco Molina-Rueda, Roberto Cano-de-la-Cuerda

**Affiliations:** 1 Multiple Sclerosis Association of Leganés. Leganés, Madrid, Spain; 2 Multiple Sclerosis Association of Fuenlabrada. Fuenlabrada, Madrid, Spain; 3 Department of Physical Therapy, Occupational Therapy, Physical Medicine and Rehabilitation, Faculty of Health Sciences, Rey Juan Carlos University, Alcorcón, Madrid, Spain

**Keywords:** Quality of life, Coordination, Multiple sclerosis, Fatigue, Muscular strength, Functionality, Rehabilitation, Powerball system

## Abstract

**Introduction:**

Although clinical and functional impairments in the lower limbs have been extensively studied in patients with MS, the upper limb (UL) are also frequently affected. Clinical impairment of the UL in patients with MS is very common with muscle strength and hand dexterity as critical factors in maintaining functional activities that are the basis for independence and quality of life in people with MS.

**Objective:**

To investigate the effects of a training protocol using the Powerball® system in combination with conventional physiotherapy on muscle strength, coordination, fatigue, functionality, and quality of life in persons with MS over an 8-week period.

**Materials and methods:**

A double-blind randomized controlled trial was conducted. The control group received conventional treatment, while the experimental group received additional UL training using the Powerball® system. Both groups received the same number of sessions and weeks of intervention. The following outcome measures were used: isometric grip and pinch strength, Box and Block Test (BBT), Nine Hole Peg Test (NHPT), Abilhand scale, Fatigue Severity Scale (FSS), Multiple Sclerosis Impact Scale (MSIS-29), and Likert satisfaction questionnaire for the experimental group. All measures were administered at baseline, after the treatment, and during a 3-week follow-up period.

**Results:**

25 patients completed the study (12 in the experimental and 13 in the control group). The experimental group showed significant improvements in coordination and manual dexterity of the more affected UL as measured by the BBT comparing pre- to post-treatment (*p* = 0.048) and pre-treatment to follow-up (*p* = 0.001), and on the less affected UP comparing pre-treatment to follow-up (*p* < 0.001) and post-treatment to follow-up (*p* = 0.034). The Likert-type satisfaction questionnaire obtained a mean score of 89.10 (± 8.54) out of 100 points.

**Conclusions:**

Upper limb treatment protocol using the Powerball® system, in combination with conventional physiotherapy for 8 weeks resulted in significant improvements in the intra-group analysis for UL coordination and manual dexterity in favor of the experimental group. The experimental group showed excellent satisfaction to the treatment.

## Highlights

Powerball® can be used for upper limb rehabilitation in persons with multiple sclerosis.

Coordination and manual dexterity may be improved after a protocol treatment with the Powerball®.

Persons with multiple sclerosis showed excellent satisfaction after a protocol treatment with the Powerball®.

## Introduction

Multiple sclerosis (MS) is a chronic, neurodegenerative, and inflammatory disease of unknown etiology that primarily affects the myelin in the central nervous system (CNS). Its main histopathological feature is the formation of plaques [1–3]. When these lesions occur, in addition to myelin involvement in multiple areas, there may be oligodendrocyte loss, gliosis and scattered axonal injury throughout the CNS, with a certain predilection for the optic nerves, brainstem, spinal cord, cerebellum and periventricular white matter [4]. MS is the leading cause of non-traumatic disability in young adults in Europe and North America reaching 2.8 million affected worldwide [5, 6]. Symptoms most commonly appear between the second and fourth decades of life [4].

MS is characterized by a wide range of symptoms and disease progression patterns, with a significant potential impact on the quality of life of individuals, as it can affect various aspects of life and have socio-economic consequences [1].

While the clinical and functional impairment of the lower limbs in people with MS has been extensively studied, the upper limbs (UL) are also commonly affected. This can include ataxia, spasticity, sensory changes and/or loss of strength [1]. In a study conducted by Kister et al. [7], involving a sample size over 20,000 subjects diagnosed with MS, highlighted the high prevalence of hand motor function disorders. These were observed in 60% of individuals with MS from the initial stages of the disease and exhibited an escalating incidence with disease progression. Several authors [8, 9] have suggested that impairment of muscle strength and manual dexterity in the ULs is directly related to activities of daily living (ADLs), which are closely associated with independence and quality of life in people with MS. Lamers et al. [10] indicated that strength is the most important variable for performing ADLs in people with MS. Cattaneo et al. [11] found that manual dexterity is crucial for performing household tasks and that the presence of participation limitations is associated with a higher predisposition to developing cognitive deficits, which are present in 50% of patients.

According to the study by Severijins et al. [12], people with MS who scored less than 6 on the Expanded Disability Status Scale (EDSS) had lower maximum grip strength and increased fatigue during static contractions compared with healthy people. Solaro et al. [13] found a correlation between isometric hand grip strength, the Block and Box Test (BBT) score, and time to perform the Nine Hole Peg Test (NHPT) with time since disease onset. They also found that the BBT score and NHPT time were correlated with the EDSS score.

Considering all these data, the high percentage of upper limb involvement in people with MS is evident, as both muscle strength and manual dexterity are reduced in people with MS, significantly affecting functionality and quality of life. In this context, the PowerBall® system [14, 15], based on the principle of a gyroscope, has been developed to strengthen the upper extremity and has shown positive results in increasing grip strength and reducing non-specific wrist pain. The device consists of a sphere containing a 200-gram rotor with an eccentric mass located 2 cm from its axis. The inner cylinder rotates around a vertical axis, creating a centrifugal force. The gyroscope is accelerated by the rotation of the wrist and the user activates the internal rotation of the device. As the rotation of the wrist increases, so does the speed of the gyroscope and the centrifugal force, requiring the user to control the rotation of the gyroscope using muscle control. To the best of our knowledge, there is no previous research that has evaluated the clinical effects of the PowerBall® system in any type of neurological disorder, so the relevance of this study in the context of UL rehabilitation in people with MS is justified.

### Aim

The purpose of this study is to investigate the effects of the Powerball® system, in combination with a conventional physiotherapy intervention, on muscle strength, coordination, fatigue, functionality, and quality of life in people with MS.

## Methods

### Design

A double blinded randomized controlled trial (RCT) (NCT05895734) was conducted according to the recommendations of the CONSORT guidelines [16]. Participants were randomly assigned to either the control group or the experimental group by a blinded investigator who was not involved in the intervention. The randomization process was performed using the Research Randomizer Version 4.0 program. The control group received conventional physiotherapy treatment, while the experimental group received the same conventional physiotherapy treatment plus treatment with the Powerball® system. All outcome measures in both groups were evaluated by blinded assessors to the intervention.

The present study received ethical approval from the Human Ethics Committee of the Rey Juan Carlos University.

### Participants

All subjects in the present study were recruited from two multiple sclerosis patient associations in the Community of Madrid (ALEM and AFEM, Madrid, Spain). Participants in this study fulfilled the following inclusion criteria: between 20 and 70 years of age, diagnosed with multiple sclerosis according to the McDonald criteria [17] with a disease duration of more than two years; EDSS score between 2 (minimal disability in one functional system) and 7 (inability to walk more than 5 m, even with assistance, limited to wheelchair mobility, although able to propel oneself and transfer without assistance; active in wheelchair for at least 12 h per day); stable medical treatment for at least six months prior to the intervention; upper limb muscle tone not greater than 2 points (moderate hypertonia, increased muscle tone during the majority of the range of motion but can be moved passively with ease in the affected limb) on the modified Ashworth scale [18, 19]; upper limb muscle balance equal or greater than 3; a score of 4 or less on the “Pyramidal Function” section of the EDSS functional scale; no cognitive impairment; ability to understand instructions and score of 4 or greater on the Mini-Mental State Examination; and a score of 2 or less on the “Mental Functions” section of the EDSS.

Exclusion criteria: diagnosis of any neurological disease or musculoskeletal disease other than multiple sclerosis; diagnosis of any cardiovascular, respiratory, or metabolic disease or other condition that might interfere with this study; history of exacerbation or hospitalization within the three months prior to the start of the assessment protocol or during the therapeutic intervention process; history of botulinum toxin treatment within the six months prior to the start of the study; presence of uncorrected visual impairment.

All patients voluntarily signed an informed consent form to participate in the study.

### Intervention

Participants in the control group received conventional physiotherapy treatment, while participants in the experimental group received conventional physiotherapy treatment plus treatment using the PowerBall® system. Both groups received 45 min of intervention per session and the same total number of sessions.

The conventional physiotherapy treatment consisted of a total of 16 sessions, with 2 sessions per week, over a period of 8 weeks. The treatment consisted of the following components [20–22]: osteokinematic mobilizations of the shoulder, elbow, and wrist joints; training activities targeting upper limb manipulative and functional skills for activities of daily living; an upper limb strengthening program, followed by a stretching program for the involved muscles. Each session lasted 45 min.

**Fig. 1:**
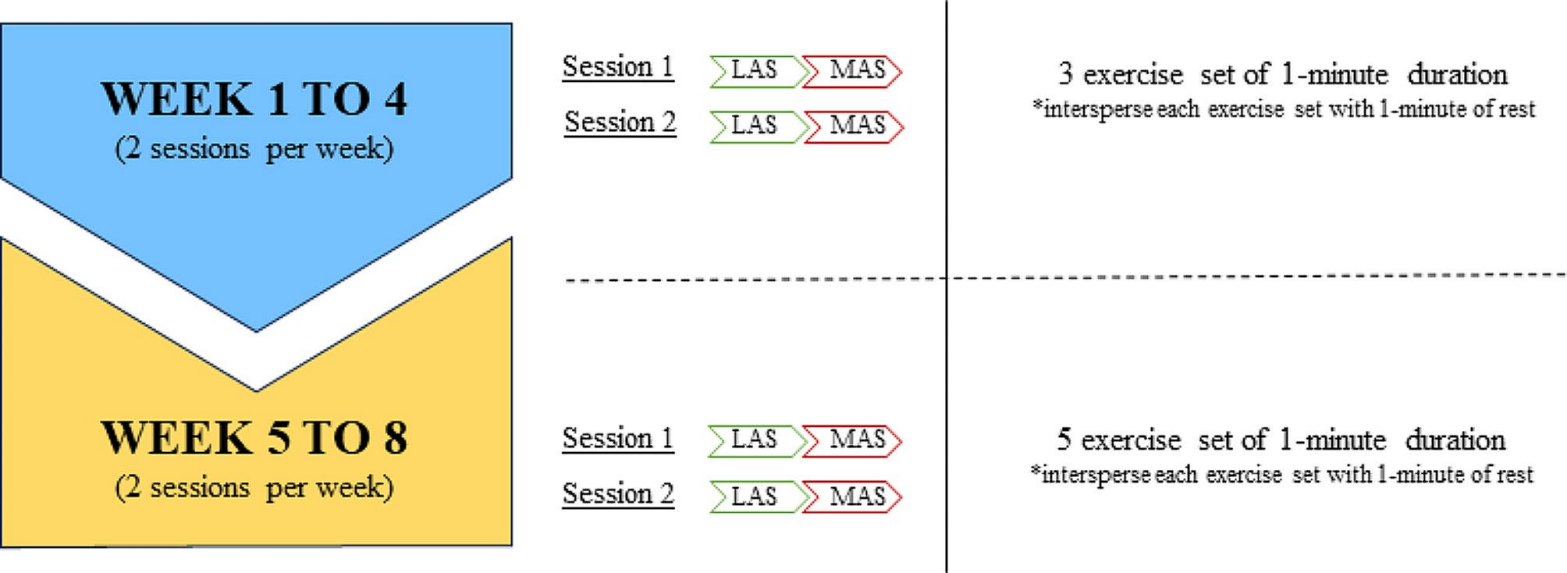
Protocol for intervention with the Powerball® system *LAS* Less Affected Side; *MAS* More Affected Side * In each of the sessions, the exercises are performed first with the less affected side and then with the more affected side

The experimental group received intervention with the PowerBall® 250 Hz gyroscope system in addition to conventional physiotherapy. The intervention consisted of 3 sets of 1-minute duration with each hand during the first 4 weeks, and 5 sets with each hand in subsequent sessions until the end of the study. The intervention always started with the dominant hand or the less affected hand (Fig. 1).

**Fig. 2:**
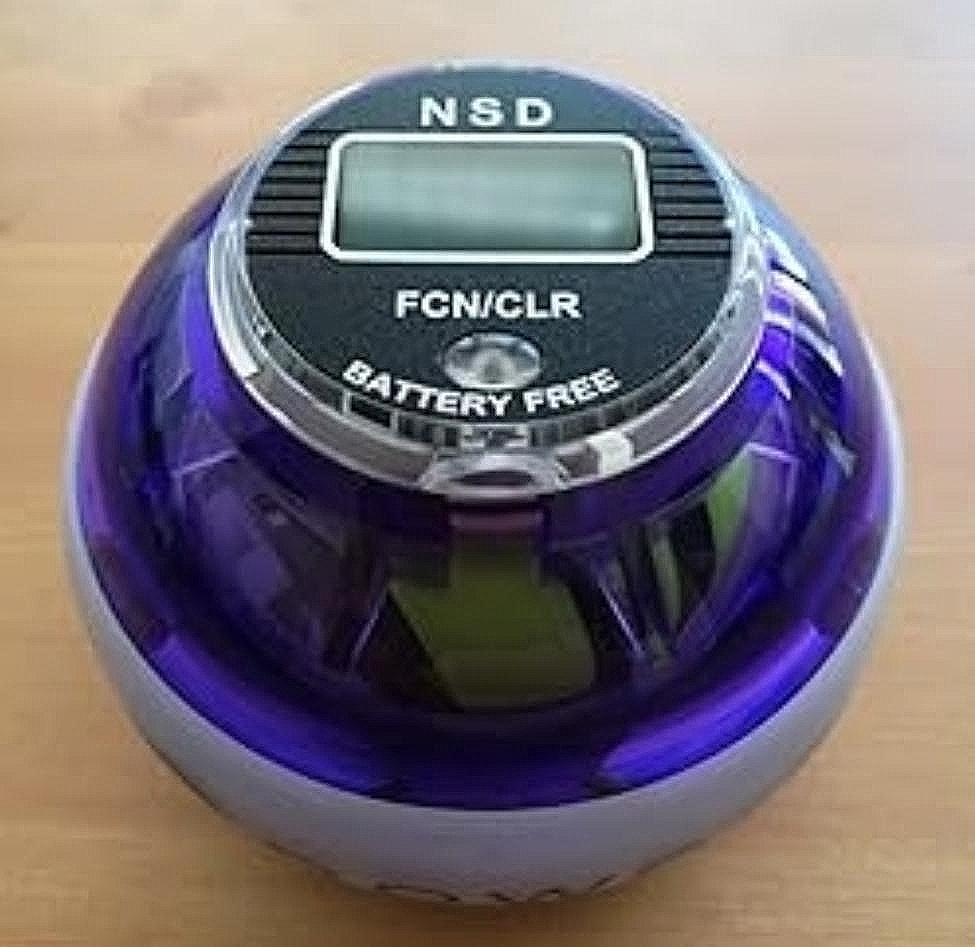
PowerBall system

As there are different models of PowerBall® and forms to initiate the gyroscope system, the manual activation model with a frequency of 250 Hz was used in this study (Fig. 2).

During the sessions with the PowerBall®, the physiotherapist was responsible for activating the device and initiating the rotation of the system. During all sessions with the device, the patient was seated upright in a chair with a backrest and no armrests to avoid interfering with the position of the working upper limb. The exercise was performed with both upper limbs, alternating between the more affected and less affected upper limb (the more/less affected side was determined by their neurologist based in their EDSS and physical exploration). The rotation of gyroscope was clockwise for the right upper limb and counterclockwise for the left upper limb. The upper limb performing the exercise was supported on a table, with the elbow flexed at 90 degrees, the shoulder flexed at 20 degrees, and the shoulder abducted at 30 degrees. The wrist was in slight extension, with forearm pronation and slight radial deviation, maintaining a comfortable position throughout the exercise. The non-exercising upper limb was placed in the patient’s lap.

Prior to the start of the sessions, a demonstration session of the device was given to familiarize the patients with the PowerBall® system.

All interventions considered the level of fatigue experienced by the patient, allowing for longer rest intervals if fatigue exceeded a score of 7 on the Modified Borg Scale [22].

### Outcomes measures

The following assessment tools were used at the beginning and end of the intervention in both groups by researchers who were not involved in the intervention. Patients were then reassessed 3 weeks after stopping Powerball® but continuing with conventional physiotherapy. Isometric Grip Strength: The JAMAR® hand dynamometer was used to measure grip strength. The JAMAR® dynamometer is widely recognized as the gold standard for grip strength assessment [23, 24]. It has demonstrated excellent test-retest and inter-rater reliability across different populations [24, 25]. The patient held the dynamometer with the upper limb in 0° abduction and rotation, the elbow flexed at 90°, and the forearm in a neutral position. The wrist was held between 0 and 15° of radial deviation, and a maximum grip was exerted for 3 s using all five fingers. The force exerted was measured in kilograms [25]. Three measurements were taken with each hand, always starting with the dominant hand, and the average of the three measurements was calculated [25].

Isometric Pinch Strength: A Baseline Pinch Gauge® was used to assess three different types of pinch grip. The two-finger opposition grip [26] in which the anterior and distal surfaces of the first finger contact the anterior surface of the pinch gauge and the anterior and distal surfaces of the second finger contact the posterior surface of the pinch gauge; the lateral pinch grip [27] in which the anterior surface of the distal phalanx of the first finger contacts the anterior surface of the pinch gauge, and the radial edge of the second finger contacts the posterior surface of the pinch gauge; and three-finger pinch grip [28] in which the anterior and distal surfaces of the first finger contact the posterior surface of the pinch gauge, and the anterior and distal surfaces of the second and third fingers contact the anterior surface of the pinch gauge. Three maximum force pinches were performed for 3 s each, alternating between the dominant and non-dominant hand. The force obtained was measured with the pinch gauge in kilograms and the average of the three results was calculated.

Box and Block Test (BBT): This test was designed to assess manual coordination and dexterity. The patient is seated in front of a wooden box divided into two identical halves containing 150 2.5 cm wooden blocks of different colors positioned in the midline. The patient’s task is to transfer as many blocks as possible from one half of the box to the other within 60 s [29]. The test is performed first with the dominant hand and then with the non-dominant hand. It is a standardized tool for measuring gross motor function of the upper limbs and has been validated for sex and age in healthy subjects [29]. It also shows low ceiling and floor effects in people with MS. Other psychometric properties, such as test-retest reliability and minimal detectable change, have been investigated in other neurological conditions and show excellent reliability and a cut-off point of 6 blocks for minimal detectable change [29].

Nine Hole Peg Test (NHPT): This test has been used to assess upper limb function, specifically fine motor skills of the hand [30]. The subject is positioned in front of a pegboard and is asked to insert and remove nine pegs, one at a time, into the nine holes of the board as quickly as possible. The dominant hand is tested first, followed by the non-dominant hand. The non-tested hand can provide stability by holding the pegboard. The score is measured in seconds and represents the time taken to complete the task. The NHPT has excellent test-retest, inter-rater, and intra-rater reliability, as well as adequate internal consistency. Scores greater than 0.27 s per pin indicate severe hand dysfunction. The minimum detectable change is 7.46 s for the non-dominant hand and 4.38 s for the dominant hand. The standard deviation of the measurement is 2.69 s for the non-dominant hand and 1.58 s for the dominant hand [30, 31].

Abilhand: It is a self-administered questionnaire designed to assess an individual’s manual dexterity, which is defined as the ability to perform a series of tasks regardless of the strategies used to perform them [32]. The questionnaire consists of 23 items that are scored on a scale of 0 to 2, corresponding to different activities of daily living (ADLs), and the patient indicates the level of difficulty experienced in performing the activities [33]. The score can range from 0 to 46, with higher scores indicating better manual dexterity. It has excellent inter- and intra-rater reliability, internal consistency, and construct validity [33].

Fatigue Severity Scale (FSS): It is a self-administered scale that assesses the severity of fatigue experienced by the patient during specific daily activities. It consists of 9 items and the scores range from 1 to 7, with a minimum score of 9 and a maximum score of 63. A higher score indicates a greater impact of fatigue on the patient’s life [34]. The standard error of measurement is 0.7 points, and the minimum detectable change is 1.9 points. Test-retest reliability is moderate, and the construct validity is excellent in people with MS [35].

Multiple Sclerosis Impact Scale (MSIS-29): It is a self-administered scale that assesses the impact of the disease on the patient’s life in the two weeks prior to administration. It consists of 29 questions, 20 of which assess the physical aspects of multiple sclerosis (MS) and 9 of which assess the psychosocial aspects. There are 5 possible answers, scored from from 1 to 5, indicating lower to higher impact of the disease. Two total scores are generated, corresponding to the physical and psychological impact subscales. The scores range from 0 to 100, with a higher score indicating a greater degree of disability [36]. The physical domain has been shown to have high internal consistency [37] and good construct validity, while the cognitive domain has good internal consistency [38]. The scale has low ceiling and floor effects, excellent Cronbach’s alpha for both parts of the scale, and a strong correlation between the physical and psychological domains [38].

Likert Satisfaction Questionnaire for the experimental group: At the end of the study, participants in the experimental group also completed a satisfaction survey about the experimental treatment using a Likert-type questionnaire developed by the research team. The questionnaire consisted of 20 items related to user satisfaction with the system used, including attributes of the tool itself, ease of use, accessibility, session design, schedules, duration, as well as attributes of the center and physiotherapist involved in the intervention. Scores could range from 1 to 5 points. The maximum possible score was 100 points, with higher scores indicating greater satisfaction.

Treatment Adherence and Adverse Effects: finally, the percentage of patients in both groups adhering to the treatment (%) and the possible occurrence of adverse effects were recorded.

### Sample size calculation

Grip strength was selected as the main outcome. The effect size of grip strength was estimated to be medium (f = 0.3). A correlation of 0.5 between repeated measures was assumed. Considering three measurements (pre-intervention, post-intervention and one month follow-up), the sphericity correction was set at 0.5. With a statistical power of 0.80 and an alpha level of 0.05, a total sample size of 20 patients was estimated. Taking into account an attrition rate of 25%, a total of 25 patients were considered necessary. Sample size was calculated using G*Power 3.1.7 software.

### Statistical analysis

All data were entered into the SPSS v.28.01 statistical package. Descriptive analysis of qualitative data was carried out using means, percentages, and ranges. The Kolmogorov-Smirnov test was used to assess the normality of the study variables. For variables with a normal distribution, a parametric analysis was performed using repeated measures analysis of variance (ANOVA) with Bonferroni post hoc adjustments. The group parameter was set as the between-subjects factor, while the within-subjects factors included each of the measurements and the treated side. A one-factor ANOVA was used to compare satisfaction and treatment attendance. Statistical analysis was performed at the 95% confidence level, with *p*-values less than 0.05 considered statistically significant.

## Results

**Fig. 3:**
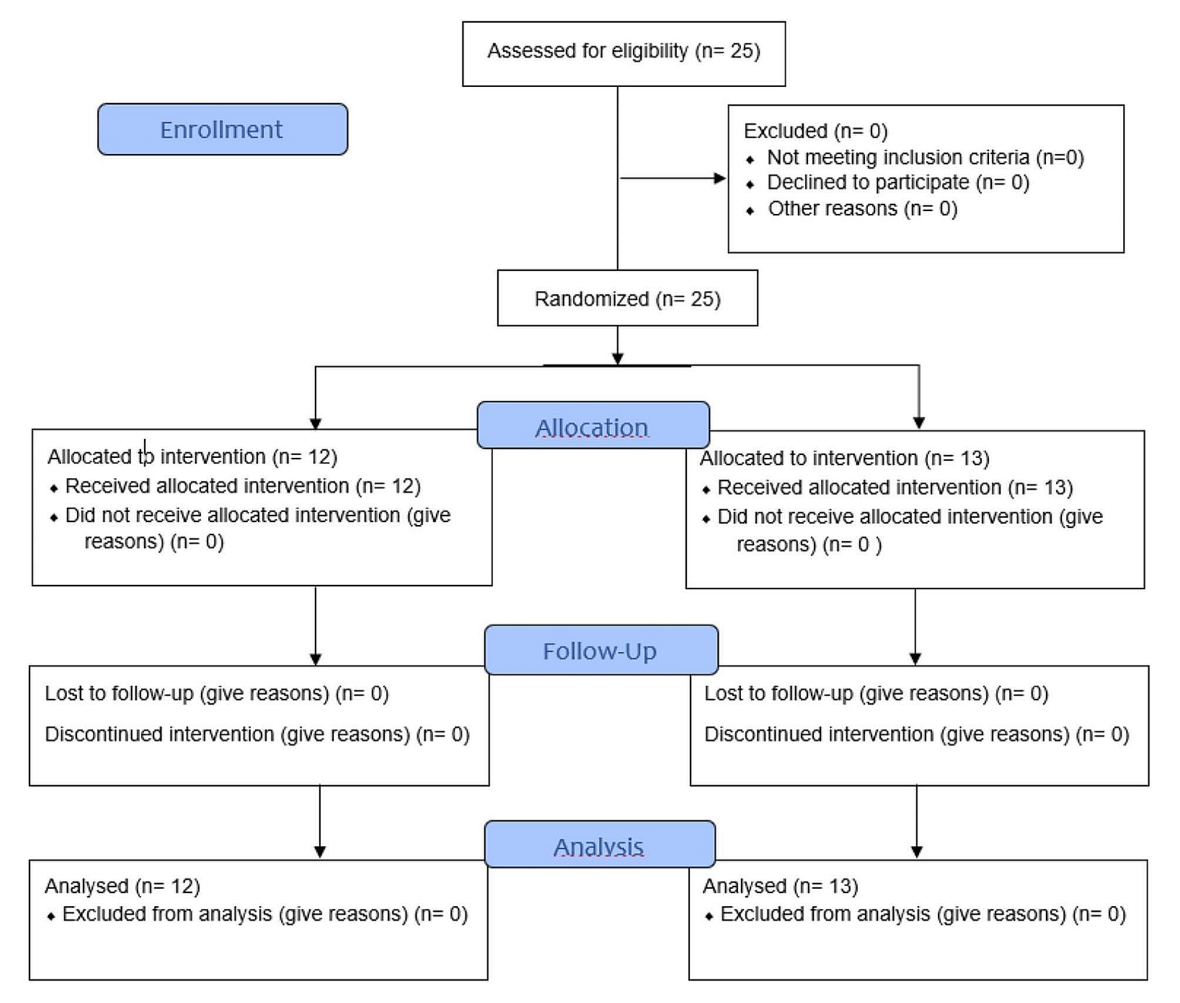
Flow chart

25 patients completed the study, with 12 subjects in the experimental group and 13 subjects in the control group. The flow chart is shown in Fig. 3.

In the experimental group, 66.6% were male and 33.4% were female, while in the control group, 46.2% were male and 53.8% were female. The mean age in the experimental group was 53.00 years (2.06), while in the control group, the mean age was 46.33 years (1.89). The mean disease duration in the experimental group was 17.75 years (3.2), while in the control group, the mean disease duration was 14 years (3.47). The remaining socio-demographic variables are shown in Table 1.

**Table 1:** Socio-demographic data of the participants

Groups (n)	Age (years) Mean (± Standard deviation)	Gender (%)		More affected side	Type of MS	Disease duration (years) Mean (± Standard deviation)	EDSS Mean (± Standard deviation)
Control group (13)	46.33 (± 1.9)		46.2 Males 53.8 Females	25% Left 75% Right	50% RRMS 50% SPMS 0% PPMS	14.00 (± 3.47)	5.55 (± 1.09)
Experimental group (12)	53.00 (± 2.06)		66.6 Males 33.4 Females	42% Left 58% Right	25% RRMS 50% SPMS 25% PPMS	17.75 (± 3.2)	4.38 (± 2.05)

*EDSS* Kurtzke Expanded Disability Status Scale; *MS* Multiple Sclerosis; *RR-MS* Relapsing-Remitting MS; *SP-MS* Secondary-Progressive MS; *PP-MS* Primary-Progressive MS

### Inter-group analysis

The ANOVA analysis showed no statistically significant differences for any of the variables analyzed in the group*side*time comparison. These results are shown in Table 2.

**Table 2:** Comparison of outcome scores (Intragroup and intergroup analysis)

Variable	Group	Pre Mean (± Standard deviation))	Post Mean (± Standard deviation)	Follow up Mean (± Standard deviation)	ANOVA (duration*side*group) *p*-value	Two-paired comparisons		
						Pre Vs. Post *p*-value	Pre Vs. Follow up *p*-value	Post Vs. Follow up *p*-value
JAMAR® more affected side	Experimental	28.73 (± 13.03)	29.39 (± 13.38)	30.38 (± 14.18)	0.859	> 0.999	0.572	0.692
	Control	23.00 (± 13.60)	24.39 (± 13.69)	23.40 (± 13.14)		0.701	> 0.999	0.638
JAMAR® less affected side	Experimental	31.60 (± 13.00)	31.90 (± 14.64)	32.75 (± 15.3)		> 0.999	> 0.999	> 0.999
	Control	24.71 (± 12.10)	26.24 (± 12.20)	24.13 (± 12.33)		0.615	> 0.999	0.213
Two-finger opposition grip more affected side	Experimental	4.6 (± 1.92)	4.03 (± 1.83)	3.91 (± 1.80)	0.1	0.172	0.180	> 0.999
	Control	3.47 (± 0.98)	4.72 (± 1y.89)	4.08 (± 1.24)		**0.017**	0.669	0.420
Two-finger opposition grip less affected side	Experimental	4.48 (± 1.31)	4.38 (± 1.36)	4.62 (± 2.42)		> 0.999	> 0.999	> 0.999
	Control	4.30 (± 1.25)	4.74 (± 1.55)	4.61 (± 0.95)		0.804	> 0.999	> 0.999
Lateral pinch grip more affected side	Experimental	7.70 (± 2.22)	7.76 (± 3.27)	7.91 (± 3.24)	0.214	> 0.999	> 0.999	> 0.999
	Control	5.47 (± 2.25)	6.58 (± 2.69)	6.86 (± 2.56)		0.431	0.393	> 0.999
Lateral pinch grip less affected side	Experimental	7.64 (± 2.41)	7.61 (± 3.20)	8.06 (± 3.26)		> 0.999	> 0.999	0.666
	Control	7.05 (± 3.08)	7.08 (± 2.77)	7.52 (± 2.87)		> 0.999	> 0.999	> 0.999
Three-finger pinch grip More affected	Experimental	6.41 (± 2.50)	6.15 (± 3.05)	6.19 (± 2.72)	0.123	> 0.999	> 0.999	> 0.999
	Control	5.22(± 2.14)	5.80 (± 1.81)	6.08 (± 2.42)		> 0.999	0.329	> 0.999
Three-finger pinch grip Less affected	Experimental	6.14 (± 1.98)	6.38 (± 2.46)	7.02 (± 2.63)		> 0.999	0.473	0.734
	Control	6.47 (± 1.96)	6.44 (± 1.76)	7.33 (± 3.06)		> 0.999	0.959	0.757
BBT More affected	Experimental	44.50 (± 7.65)	49.17 (± 10.15)	52.08 (± 9.53)	0.338	**0.048**	**0.001**	0.380
	Control	39.23 (± 10.20)	40.77 (± 10.89)	45.08 (± 11.55)		> 0.999	0.069	**0.005**
BBT Less affected	Experimental	47.75 (± 9.55)	49.92 (± 11.97)	54.83 (± 9.93)		0.312	**< 0.001**	**0.034**
	Control	41.69 (± 10.61)	42.46 (± 11.24)	45.46 (± 10.38)		> 0.999	**0.012**	0.282
NHPT More affected	Experimental	34.65 (± 17.13)	29.86 (± 11.04)	33.45 (± 23.30)	0.765	0.077	> 0.999	0.629
	Control	37.86 (± 18.17)	35.36 (± 13.64)	35.46 (± 15.49)		0.623	0.503	> 0.999
NHPT Less affected	Experimental	28.16 (± 3.45)	28.18 (± 6.29)	26.72 (± 6.08)		> 0.999	> 0.999	> 0.999
	Control	34.61 (± 20.84)	35.30 (± 19.07)	33.31 (± 13.73)		> 0.999	> 0.999	0.826
FSS	Experimental	49.00 (± 11.83)	45.67 (± 8.37)	48.58 (± 9.25)	0.531	0.244	> 0.999	0.607
	Control	38.31 (± 19.24)	37.62 (± 17.76)	38.15 (± 18.72)		> 0.999	> 0.999	> 0.999
ABILHAND	Experimental	35.42 (± 8.43)	35.67 (± 8.46)	36.75 (± 8.50)	0.859	> 0.999	> 0.999	0.992
	Control	34.62 (± 12.03)	36.15 (± 9.82)	37.23 (± 8.84)		> 0.999	0.485	0.943
MSIS-29 physical score	Experimental	57.07 (± 20.53)	64.42 (± 18.52)	62.29 (± 16.62)	0.504	0.332	0.695	0.955
	Control	43.75 (± 31.66)	43.95 (± 29.45)	43.85 (± 29.02)		> 0.999	> 0.999	> 0.999
MSIS-29 psychological score	Experimental	47.96 (± 25.18)	50.26 (± 22.84)	58.02 (± 20.63)	0.451	> 0.999	0.146	0.438
	Control	44.53 (± 29.87)	43.68 (± 33.91)	46.22 (± 33.78)		> 0.999	> 0.999	> 0.999

*BBT* Box and Block Test; *NHPT* Nine Hole Peg Test; *FSS* Fatigue Severity Scale; *MSIS-29* Multiple Sclerosis Impact Scale. All *p-*values present Bonferroni adjustment

The mean adherence rate recorded in the experimental group was 100%, while in the control group it was 87.96%, with statistically significant differences in favor of the experimental group (*p* = 0.002).

### Within-group analysis

Significant changes were observed in the within-group analysis of coordination and dexterity evaluated through the BBT (Box and Block Test). In the experimental group, there were significant differences between pre-treatment and post-treatment comparisons (*p* = 0.048) and between pre-treatment and follow up comparisons (*p* = 0.001) in the more affected side. In the less affected side of the experimental group, significant differences were found between pre-treatment and follow-up comparisons (*p* = < 0.001) and between post-treatment and follow-up comparisons (*p* = 0.034). In addition, the control group also showed significant changes between post-treatment and follow-up comparisons in the more affected side (*p* = 0.005), and between pre-treatment and follow-up comparisons in the less affected side (*p* = 0.012).

For the remaining variables, no significant within-group changes were observed across the three measures in either the experimental or control groups. Further details of the results of the between-group analysis are given in Table 2.

The Likert-type satisfaction questionnaire administered to the experimental group obtained an average score of 89.10 (8.54) out of 100 points. Finally, no adverse effects were reported in any participant of the experimental group.

## Discusion

The results of this study showed that the combination of a conventional physiotherapy program with upper limb strength training using the Powerball® system resulted in statistically significant within-group changes in both coordination and dexterity of both the more affected upper limb and the less affected upper limb. Participants reported excellent satisfaction and high adherence to the treatment. However, no effects were observed on hand and grip muscle strength, fatigue, functionality, and quality of life in people with multiple sclerosis (MS) with an EDSS score of 2 to 7 points compared with a control group receiving conventional physiotherapy.

In our opinion, the positive effects on coordination and dexterity observed in our study can be attributed to the specific nature of the device actively used, which requires continuous concentric isotonic contractions of the forearm and wrist muscles during the time of the protocol used. The coordinated gestures of the forearm and wrist muscles to rotate the device were encouraged during the sessions by the physiotherapist in charge of the protocol. In other words, we believe that the changes in coordination and dexterity were a result of the specific trained task to use the Powerball® system.

The results obtained in the present study in terms of muscle strength, fatigue and quality of life can be attributed to the design of the proposed protocol as well as to the inherent nature of the disease. It may also be due to the difference in the percentage of disease type between the groups, as the experimental group included individuals with a higher percentage of progressive types of the disease, or even due to the presence of more men in the experimental group who have a worse prognosis than women. In this regard, previous research has identified the different variables that influence the strength training prescription.

### Training intensity

Helms et al. [39] define intensity as the amount of weight lifted, usually measured as a percentage of 1RM (the weight at which a complete movement can only be performed), or as the proximity to this maximum effort, typically defined by a value on the Rating of Perceived Exertion (RPE) scale. Among the various tools used to measure intensity, the percentage of 1RM stands out. This percentage can be calculated in a number of ways, for example by performing an “As Many Reps As Possible” (AMRAP) set or by performing an actual 1RM test. The main disadvantage of this system is that it is not appropriate to perform a 1RM test for all the movements performed during training or, in this case, during physiotherapy.

Another tool to measure intensity would be the number of maximum repetitions (RM), where exercise load is defined as the maximum number of repetitions that can be performed with a given weight. In the context of our research, the Power-Ball® system represents a mechanism similar to isoinertial pulleys, with both devices providing resistance proportional to the force applied by the subject. While the latter has been shown to improve strength and neuromuscular adaptations in healthy individuals, as well as neuromuscular function and exercise capacity in stroke survivors [40], the design of the Power-Ball® system used in our study did not allow the exercise to be controlled at a defined intensity in any of the aforementioned terms, so the intensity at which it is performed remains unknown.

According to the Arnold-Schultz law or threshold law [41], any stimulus must reach a minimum intensity to induce adaptations in the body, just as it should not exceed a threshold to avoid exceeding the body’s ability to adapt. Taking this into account, despite the potential therapeutic value of the tool under study, our protocol may have resulted in subjects not reaching the minimum intensity threshold to induce the relevant adaptations in our patients.

### Training volume

Training volume, on the other hand, is defined as a measure of the total amount of exercise performed [39]. It can be quantified on the basis of the total number of repetitions performed, the total weight lifted, or the duration of the session or training period. Currently, it is most common to quantify weekly sets or effective repetitions (repetitions performed at a minimum intensity threshold to produce adaptations) as these are the easiest to quantify. According to Helms et al. [39], the appropriate weekly volume for strength gains should be between 5 and 12 sets per muscle group. However, several studies [42, 43] have shown beneficial effects in different domains in people with MS with lower training volumes of 4 to 6 sets per week, divided into several weekly sessions. Although the workload proposed in the design of this study is consistent with the workload recommended in other research for people with MS, the lack of proper intensity monitoring may have resulted in some of the workload not being performed within the aforementioned threshold volumes, potentially reducing the beneficial effects of training with the PowerBall® system.

### Training frequency

Helms et al. [44] define frequency as the variable that distributes and organizes volume and intensity over a period of training. In general, if exercise intensity is maintained within appropriate thresholds, performing a given volume spread over several weekly sessions will produce better results than the same volume concentrated in a single session. In our study, we found significant within-group changes in coordination and dexterity assessed by the BBT in favor of the experimental group, possibly due to the rhythmic and coordinated work of mobilizing the rotor of the Powerball® system for both the more and less affected sides. However, spreading the weekly volume over 3 days, combined with training at optimal intensity, would probably have improved the results of this study for the other outcome measures, particularly muscle strength.

### Training rest periods

Finally, another variable in exercise prescription is rest periods. Various authors suggest that rest periods between sets should be between 2 and 3 min [44] and may be as long as 5 min in healthy individuals. Reducing the duration of these rest periods may result in less oxygen being delivered to the tissues, leading to a greater degree of fatigue, which will limit the ability to maintain an appropriate intensity level in subsequent sets. On the other hand, other research suggests that the type of practice is the most important factor in motor skill retraining [45], where practice refers to any activity specifically designed to improve current levels of performance. There are different types of practice, with the main distinction being between intensive practice and distributed practice, with the former involving more work than rest, and the latter involving more rest than work [46]. Distributed practice appears to have a greater effect on learning, whereas intensive practice may lead to fatigue, which should be avoided in the context of patients with MS. Several studies [43] have emphasized the need to include both strength and aerobic training and to appropriately manage symptoms such as fatigue or heat sensitivity by making necessary modifications to the therapeutic approach in patients with MS. In our protocol, we attempted to avoid the onset of fatigue using the modified Borg scale and to ensure that rest periods were sufficiently long to prevent adverse effects due to poor fatigue management. However, rest periods equal to or longer than 3 min were not implemented. Therefore, although the exercises were not performed in a fatigued state, it is possible that due to the design of the protocol, optimal rest between sets was not achieved in the sessions and/or some participants did not achieve maximal intensity, limiting potential gains in muscle strength.

### Training characteristics in people with MS

Several studies [42, 43, 47–49] have extensively analyzed the effects of aerobic and anaerobic exercise in people with MS, demonstrating that isolated or combined resistance training has beneficial effects in patients with MS. The main characteristics of the protocols analyzed are as follows [43, 44, 47–49]: the prescribed intensity ranged from 35 to 90% of the subject’s 1RM, while in some studies it followed a pattern of 10–12 repetitions with a progressively increasing load approaching the subject’s 1RM. However, it should be noted that a significant number of trials did not report the intensity used. The volume per session consisted of 2–3 sets per exercise in each session, with a weekly frequency of 2–3 sessions. Rest periods between sets ranged from 1 to 5 min, with 2–3 min being the most common range. The duration of the protocol varied from 8 to 26 weeks, with the majority falling between 10 and 12 weeks. Studies that focused exclusively on the upper limbs followed a similar pattern, but with the peculiarity of increasing the session time from 45 to 60 min or using exercises with loads far from 1RM but with a high number of repetitions.

The protocol presented in our research differed from the previously described recommendations for producing changes in outcome measures in patients with MS, specifically the lack of measurement of applied intensity, insufficient frequency per week, inadequate rest periods between sets, insufficient duration of the protocol, as well as the temporal extension of the sessions. However, these deviations were accepted due to organizational and time constraints in the context of two patient associations.

### Training adherence and satisfaction

It is worth noting that the level of participation was very high in both groups, especially in the experimental group with a 100% participation rate. In terms of satisfaction, the results of the Likert-type questionnaire showed excellent satisfaction with the experimental approach, with an average score of 89.10 out of 100. The PowerBall® system added dynamism to conventional treatment without causing any adverse effects in the experimental group. It should also be considered that all patients recruited for this study had a range of disability in the EDSS, so the experimental protocol could contribute to maintaining stability at a clinical level for the analysed variables, which is also a positive outcome in a degenerative and progressive condition like MS. However, it is important to notice that our protocol was always supervised by a health professional, so we cannot extrapolate our findings to an unsupervised home-based treatment. Future studies should investigate these aspects.

### Limitations

There are several limitations to our study. First, the results obtained in this study should not be extrapolated to all patients with MS or patients with other neurological diseases. These findings should be interpreted with caution, especially in subjects with different disease duration, different EDSS scores, or more severe upper limb impairment. In addition, the sampling method used may have influenced the results, as the study was conducted on a population of patients with MS from MS associations. In addition, future studies should recruit a larger number of patients with more pronounced upper limb weakness. Finally, future randomized controlled trials should compare the Powerball® system with conventional treatment, taking into account the specifications of the protocol used.

## Conclusions

The Powerball® system, in combination with a conventional physiotherapy program, produced statistically significant within-group improvements in coordination, both in the upper limbs, with excellent satisfaction and treatment adherence in people with MS with an EDSS between 2 and 7. Nevertheless, there was no difference with the control group. The Powerball® system did not produce effects on grip and pinch strength, fatigue, functionality, and quality of life within-group or inter-group. Future studies should confirm our findings with larger sample sizes and considering the specifications of the protocol used.

## Data Availability

All the data and materials could be found at Faculty of Health Sciences of Rey Juan Carlos University.
